# Meta-analysis of the effects of available phosphorus levels and phytase on eggshell quality parameters in laying hens: influence of dose, enzyme origin, and nutritional reduction

**DOI:** 10.1016/j.psj.2026.107283

**Published:** 2026-06-12

**Authors:** Adiel Vieira de Lima, Matheus Ramalho de Lima, Amanda Itoh de Medeiros, José Henrique Stringhini, Thácyla Beatriz Duarte Correia, Apolônio Gomes Ribeiro, Ilaiane Barbosa Matias Barros, Luayne Morais Correa, Jamilly Lima Ferreira Oliveira, Janete Gouveia de Souza, Ricardo Romão Guerra, Lucas Rannier Ribeiro Antonino Carvalho, Fernando Guilherme Perazzo Costa

**Affiliations:** a Department of Animal Science, Federal University of Paraíba, Areia, Paraíba, Brazil; b Department of Animal Sciences, Federal Rural University of the Semi-Arid Region, Mossoró, Rio Grande do Norte, Brazil; c Department of Veterinary Sciences, Federal University of Paraíba, Areia, Paraíba, Brazil; d School of Veterinary and Animal Science, Federal University of Goiás, Goiânia, GO, Brazil; e Academic Unit of Serra Talhada, Federal Rural University of Pernambuco (UFRPE-UAST), Serra Talhada, Pernambuco, Brazil; f Federal University of Agreste of Pernambuco (UFAPE), Garanhuns, Pernambuco, Brazil; g Academic Unit of Specialized Agricultural Sciences (UAECIA), Macaíba Campus, Federal University of Rio Grande do Norte (UFRN), Macaíba, Rio Grande do Norte, Brazil; h Department of Physiology and Pharmacology, Karolinska Institutet, Biomedicum 5B, Solnavägen 9, Stockholm S-171 77, Sweden

**Keywords:** Mineral availability, Eggshell strength, Enzyme supplementation, Nutrient digestibility, Calcium metabolism

## Abstract

This study aimed to evaluate, through a meta-analysis, the effects of phytase supplementation in diets for laying hens on eggshell quality parameters, including shell weight percentage (SWP), shell thickness (ST), shell breaking strength (SBS), and specific gravity (SG). The methodology consisted of a systematic review and quantitative integration of data from 16 scientific articles published between 2021 and 2025, analyzing statistical contrasts among diets with adequate phosphorus (P) levels (positive control), phosphorus-deficient diets (negative control), and diets supplemented with the enzyme phytase. The main results showed that dietary phosphorus reduction significantly impaired eggshell structural quality, reducing ST by an average of 3.50 µm and SBS by 0.23 kgf, whereas SWP and SG were not markedly affected. Phytase supplementation effectively mitigated these adverse effects, increasing ST by an average of 3.14 µm and SBS by 0.20 kgf compared to the deficient group, restoring these parameters to levels comparable to those observed in hens fed nutritionally adequate diets. Additionally, the enzyme’s efficacy remained stable regardless of its origin (bacterial or fungal) or the evaluated dosage (up to 1,000 FTU/kg). It is concluded that phytase is a key nutritional tool for maintaining eggshell structural quality under conditions of phosphorus deficiency, optimizing mineral utilization and preserving the productive efficiency of laying hens.

## Introduction

Global egg production has shown steady growth over recent decades, establishing itself as one of the most important sectors of poultry production due to the high nutritional value of eggs and the excellent productive efficiency of laying hens. In this context, eggshell quality is considered one of the main factors determining the economic efficiency of egg production, as it directly influences losses during egg collection, grading, transportation, and storage (Hincke et al., 2012; Montenegro et al., 2019). Fragile shells or shells with poor structural integrity increase the occurrence of cracked or broken eggs, leading to economic losses and a higher risk of microbial contamination, thereby compromising food quality and safety (Nys et al., 2004; Roberts, 2004). Eggshell quality is commonly assessed by parameters such as shell thickness (ST), shell breaking strength (SBS), specific gravity (SG), and shell weight percentage (SWP), which reflect its structural integrity (Roberts, 2004; Hincke et al., 2012).

Eggshell formation occurs primarily in the uterus, also known as the shell gland, which is the final stage of egg formation in the oviduct of laying hens (Eltahan et al., 2023; Waters et al., 2024). During this period, there is intense deposition of calcium carbonate (CaCO₃) onto the previously formed shell membranes, resulting in a rigid structure responsible for protecting the internal contents of the egg (Waters et al., 2024, 2025). This process depends directly on the mineral metabolism of the birds, especially the availability of calcium (Ca) and phosphorus (P), which are essential for proper eggshell mineralization (Eltahan et al., 2023; Waters et al., 2025). Calcium is supplied both through dietary absorption and mobilization of medullary bone reserves, highlighting the importance of nutritional and physiological factors in shell formation (Eltahan et al., 2023; Waters et al., 2024, 2025).

Phosphorus is an essential and vital nutrient for laying hens, playing a fundamental role in bone metabolism, skeletal mineralization, and the maintenance of egg production (Javadi et al., 2021; Eltahan et al., 2023; Park et al., 2024). However, a major nutritional challenge is that approximately two-thirds of the total phosphorus present in plant-based feed ingredients is bound in the form of phytate (Jing et al., 2021; Moura et al., 2023), a compound with limited availability to poultry due to the low activity or absence of endogenous phytase in their gastrointestinal tract (Saleh et al., 2021; Pirzado et al., 2024; Sacakli et al., 2025). Low phosphorus availability may result in reduced bone mineralization and increased mobilization of skeletal mineral reserves to sustain egg production (Javadi et al., 2021; Waters et al., 2025), favoring metabolic disorders such as osteoporosis and cage layer fatigue (Lima et al., 2024; Waters et al., 2025). In addition, phosphorus deficiency may impair eggshell quality, increasing the incidence of cracked, broken, or shell-less eggs (Roque et al., 2023; Sacakli et al., 2025), and reduce flock productivity, negatively affecting laying rate, egg mass, and feed conversion efficiency (Sun and Kim, 2021; Moura et al., 2023; Pirzado et al., 2024).

In this context, exogenous phytase supplementation in poultry diets has been widely adopted as a nutritional strategy to increase the availability of phosphorus from plant-based ingredients by hydrolyzing the phosphoester bonds of phytate molecules (Saleh et al., 2021; Sun and Kim, 2021; Moura et al., 2023; Sacakli et al., 2025). This process promotes the release of inorganic phosphorus for intestinal absorption, while also increasing the availability of other minerals, such as calcium, zinc, manganese, iron, and magnesium, previously bound to phytate (Eltahan et al., 2023; Lima et al., 2024; Pirzado et al., 2024). Additionally, phytate degradation reduces its antinutritional effects, improving nutrient digestibility and utilization efficiency (Javadi et al., 2021; Lima et al., 2024; Sacakli et al., 2025).

Despite the well-established use of exogenous phytase in poultry nutrition, findings regarding its effects on eggshell quality remain highly variable in the scientific literature, with studies reporting positive effects, limited responses, or no significant effects (Javadi et al., 2021; Jing et al., 2021; Sacakli et al., 2025). These discrepancies may be attributed to several experimental factors, including phytase dose, dietary phosphorus and calcium levels, enzyme origin, and physiological characteristics of the birds (Sun and Kim, 2021; Sacakli et al., 2025; Pirzado et al., 2024). Differences in experimental duration and diet composition may further contribute to the variability of results (Javadi et al., 2021; Jing et al., 2021; Sun and Kim, 2021).

Given the variability of results reported in the literature, a quantitative approach capable of integrating the available evidence is warranted. In this regard, meta-analysis enables the synthesis of results from independent studies, allowing the identification of general response patterns and the estimation of the magnitude of phytase supplementation effects.

Therefore, this study aimed to evaluate, through meta-analysis, the effects of phytase supplementation in diets for laying hens on eggshell quality, considering shell weight percentage, shell thickness, shell breaking strength, and specific gravity. Additionally, the effects of moderators such as the magnitude of nutritional reduction, phytase origin, and enzyme inclusion level were investigated.

## Materials and methods

### Search strategy and study selection

A systematic literature review was conducted to identify studies evaluating the effects of phytase supplementation on eggshell quality in laying hens. Searches were performed in the electronic databases PubMed, Scopus, and Web of Science, with no restriction on year of publication. The search strategy was structured using Boolean operators (AND, OR) to combine terms related to the species, the enzyme, and the variables of interest. The following combination of keywords was used: [(chickens OR hen OR "laying hen*") AND (phytase OR phytate)].

The retrieved records were exported to EndNote software for reference management and duplicate removal. Subsequently, studies were selected through sequential screening of titles, abstracts, and full texts, based on their relevance to the objective of the meta-analysis. The processes of identification, screening, eligibility, and study inclusion followed the recommendations of the PRISMA Statement (Moher et al., 2009). The last literature search was conducted on January 26, 2026.

### Eligibility and exclusion criteria

Studies retrieved from the literature search were assessed for eligibility through sequential evaluation of titles, abstracts, and full texts according to predefined criteria.

Only *in vivo* experimental studies conducted with laying hens were included in the meta-analysis. Eligible studies had to evaluate diets supplemented with exogenous phytase, regardless of enzyme origin (fungal or bacterial), and include a control treatment without phytase supplementation, with or without a reduction in available phosphorus. In addition, studies were required to assess eggshell quality parameters and provide the statistical information necessary for analysis, including treatment means, number of experimental replicates, and measures of variability.

The variables considered in the meta-analysis included SWP (%), SBS (kgf), ST (µm), and SG (g/cm³). When these variables were reported in different units, values were converted to standardized units to ensure comparability among studies.

The eggshell quality variables included in the meta-analysis were obtained from the original studies according to standard methodologies commonly used in poultry science. Specific gravity (SG) was determined based on Archimedes’ principle, calculated as the ratio between egg weight in air and egg weight in water, with correction for water temperature. Shell weight percentage (SWP) was calculated as the ratio between dry shell weight and total egg weight, multiplied by 100. Shell thickness (ST) was measured at the equatorial region of the egg using a micrometer, typically as the average of multiple measurements per egg. Shell breaking strength (SBS) was determined by applying increasing pressure to the egg using a mechanical or digital device until shell rupture, with results expressed in kgf. These standardized definitions ensured consistency in data extraction and comparability among studies included in the meta-analysis.

Studies were excluded if they involved other species, such as broilers, quail, or breeder hens; were conducted *in vitro*; used computational models; or lacked a control group. Studies evaluating phytase in combination with other enzymes, without the possibility of isolating the effects of phytase, were also excluded. Additional exclusion criteria included unclear reporting of phytase levels or dietary nutrient reduction strategies, studies assessing only productive performance without measuring eggshell parameters, and non-primary research articles such as literature reviews, meta-analyses, book chapters, theses, dissertations, or other documents not published in peer-reviewed scientific journals. Duplicate studies identified during the screening process were also removed.

### Data extraction and organization

Data from eligible studies were extracted and organized into an electronic spreadsheet containing information on experimental characteristics, dietary composition, and the results of the evaluated variables. For each study, the following information was recorded: study identification (author and year), initial and final age of the birds, type of phytase used (fungal or bacterial), dietary calcium and available phosphorus levels, phytase dose in the diet (FTU/kg, phytase units), number of experimental replicates, number of birds per treatment, and the mean values of the evaluated variables along with their respective measures of variability.

When results were reported as standard error of the mean (SEM), standard deviation (SD) was calculated using the equation: SD = SEM × √n, where n represents the number of experimental replicates. When results were presented as coefficient of variation (CV), standard deviation was calculated using the formula: SD = (CV × mean) / 100. Values reported in different units were also converted to standardized units to ensure comparability across the included studies.

### Definition of experimental contrasts

To evaluate the effects of dietary available phosphorus reduction and phytase supplementation on eggshell quality parameters, experimental contrasts were established among the treatments identified in the studies included in the meta-analysis. Treatments were classified into three categories: positive control (PC), corresponding to diets formulated without nutrient reduction and without phytase supplementation; negative control (NC), corresponding to diets with reduced available phosphorus and without phytase supplementation; and phytase treatments, corresponding to the negative control diet supplemented with different levels of phytase. Each phytase treatment within a study was considered an independent observation in the database.

Based on this classification, three main comparisons were performed among the experimental treatments. The PC vs. NC contrast was used to evaluate the effect of dietary available phosphorus reduction on eggshell quality parameters. The NC vs. Phytase contrast was used to assess the ability of phytase supplementation to recover eggshell quality parameters in diets with reduced available phosphorus. Finally, the PC vs. Phytase contrast was used to determine whether phytase supplementation in phosphorus-reduced diets could restore the evaluated parameters to levels similar to those observed in the positive control.

### Statistical analysis and meta-analysis

Statistical analyses were performed using R software (version 4.2.2) through the meta package. The meta-analysis was conducted according to the methodological recommendations described in the Cochrane Handbook for Systematic Reviews of Interventions (Higgins et al., 2019).

For continuous variables, effect sizes were calculated using the mean difference (MD) between the compared treatments, based on the means, standard deviations, and number of eggs analyzed (experimental replicates) in each group.

Effect estimates were obtained using the inverse-variance method under a random-effects model, as variability among the included studies was expected due to experimental, nutritional, and methodological differences. Between-study variance (τ²) was estimated using the Restricted Maximum Likelihood (REML) method, and results were expressed as mean differences with 95% confidence intervals.

Heterogeneity among studies was assessed using Cochran’s Q test and quantified by the I² statistic, which represents the proportion of total variability attributable to between-study heterogeneity (Higgins et al., 2019). I² values close to 0% indicate low heterogeneity, whereas higher values indicate greater inconsistency among the results of the included studies.

The risk of bias of the included studies was assessed based on methodological criteria related to experimental design, treatment description, sample size, and reporting of variability measures, following the recommendations of the Cochrane Risk of Bias Tool (Higgins et al., 2011).

In addition to the overall analysis, subgroup analyses were performed to investigate potential sources of variation among the included studies. The subgroups considered included the type of phytase used (fungal or bacterial), the level of enzyme inclusion in the diet, and the magnitude of the reduction in available phosphorus between the compared treatments. Phytase dose was classified into three categories (≤600, 600–1,000, and >1,000 FTU/kg), whereas the difference in available phosphorus level between diets was classified into two categories (≤0.02% and >0.02%).

The application of subgroup analyses varied according to the contrast evaluated. For the PC vs. NC contrast, only the subgroup related to the difference in available phosphorus was considered, since neither treatment included phytase supplementation. For the NC vs. Phytase contrast, subgroups related to phytase type and enzyme inclusion level were evaluated. For the PC vs. Phytase contrast, all previously described subgroups were considered.

Subgroup analyses were conducted only when more than one category was available among the included studies, allowing statistical comparisons between groups. When a given variable presented only one level among the available studies, subgroup analysis was not performed.

## Results

### Study selection

The search of electronic databases initially identified 2,690 records, including 403 from PubMed, 370 from Scopus, and 1,917 from Web of Science. After the removal of 400 duplicate records, 2,290 studies remained for the screening stage. Considering the inclusion criterion related to the time frame, only studies published from 2021 onward were selected, resulting in 590 records eligible for initial evaluation.

Subsequently, titles and abstracts were screened, and 566 studies were excluded for not meeting the eligibility criteria, mainly because they did not evaluate eggshell quality parameters or did not investigate phytase supplementation in diets for laying hens. As a result, 24 studies were selected for full-text assessment.

**Fig. 1:**
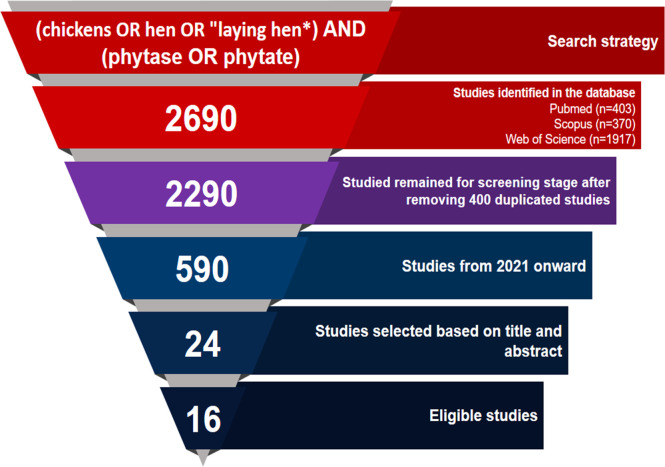
PRISMA flow diagram illustrating the process of identification, screening, and selection of studies included in the meta-analysis.

After full-text review, an additional eight studies were excluded due to the absence of an appropriate control group or lack of the statistical information required for meta-analysis. At the end of the selection process, 16 studies met all eligibility criteria and were included in the meta-analysis. The complete process of study identification, screening, eligibility, and inclusion is presented in the PRISMA flow diagram (Fig. 1).

### Characteristics of the included studies

The studies included in the meta-analysis are presented in Table 1. In total, 16 studies published between 2021 and 2025 were selected. These studies were conducted in different countries, including Brazil, South Korea, the United States, Poland, Turkey, China, Canada, Spain, Australia, and Egypt. A relatively broad geographical distribution was observed, with a higher concentration of studies conducted in Brazil and South Korea.

**Table 1:** Characteristics of the studies included in the systematic review evaluating the effects of phytase supplementation on eggshell quality parameters in laying hens.

Code	Study	Year	Country	Journal	Hen Age (weeks)	Variables Evaluated	Phytase Dose (FTU/kg)	Phytase Type	Available P (%)
1	Byczynski et al.	2025	Poland	Annals of Animal Science	45–57	SWP, ST, SBS	0, 6,575	Fungal	0.375–0.124
2	Sacakli et al.	2025	Turkey	PLOS One	23–44	ST, SBS	0, 300, 600	Bacterial	0.38–0.12
3	Waters et al.	2025	United States	Journal of Applied Poultry Research	60–80	SWP, ST, SBS	0, 400, 1,500	Bacterial	0.42–0.25
4	Lima et al.	2024	Brazil	Animals	44–64	SWP, ST, SBS, SG	0, 500, 1,000, 1,500, 3,000	Bacterial	0.49
5	Park et al.	2024	South Korea	Poultry Science	13–32	ST, SBS	0, 500	Bacterial	0.45–0.25
6	Pirzado et al.	2024	China	Animals	22–32	ST, SBS	0, 250, 1,000, 2,000	Fungal	0.34–0.14
7	Waters et al.	2024	United States	Journal of Applied Poultry Research	40–60	SWP, ST, SBS	0, 400, 1,500	Bacterial	0.44–0.27
8	Eltahan et al.	2023	South Korea	Poultry Science	73–80	ST	0, 1,000	Fungal	0.30–0.25–0.20
9	Moura et al.	2023	Brazil	Poultry Science	23–72	SWP, ST, SBS	0, 300, 600, 900	Bacterial	0.35–0.12
10	Roque et al.	2023	Brazil	Revista Colombiana de Ciencias Pecuarias	70–86	SWP, ST, SBS	0, 300, 600, 900	Bacterial	0.35–0.23–0.19–0.17
11	Farias et al.	2021	Brazil	Brazilian Journal of Poultry Science	58–107	SWP, ST, SG	0, 450, 900	Bacterial/Fungal	0.43–0.25
12	Javadi et al.	2021	Spain	Animals	22–31	ST	0, 500, 1,000	Fungal	0.44–0.20
13	Jing et al.	2021	Canada	Animal	22–34	SWP, ST, SG	0, 1,000	Bacterial	0.30–0.20
14	Ruhnke et al.	2021	Australia	Animals	19–24	SWP, ST, SG	3,500	Fungal	0.48
15	Saleh et al.	2021	Egypt	Animals	42–52	SWP, ST	5,000	Bacterial/Fungal	0.46–0.32
16	Sun & Kim	2021	South Korea	Japan Poultry Science	63–72	ST, SBS	0, 600, 1,200	Bacterial	0.12

SWP = Shell weight percentage (%); SBS = shell breaking strength (kgf); ST = shell thickness (µm); SG = egg specific gravity (g/cm³). FTU = phytase activity unit (phytase units); Available P = available phosphorus in the diet.

The age of the hens used in the experiments varied considerably among studies, ranging from 13 to 107 weeks of age, thereby encompassing different productive phases of laying hens, from the onset of lay to more advanced stages of the production cycle. Phytase doses also showed substantial variation, ranging from 0 to 6,575 FTU/kg of feed. Intermediate inclusion levels between 300 and 1,000 FTU/kg were the most frequently evaluated, although some studies also investigated phytase superdosing, with levels exceeding 1,500 FTU/kg.

Regarding the type of enzyme used, most studies evaluated bacterial phytases, followed by fungal phytases, while a few studies assessed combinations of both enzymatic sources. Concerning the composition of the experimental diets, available phosphorus (Available P) levels ranged approximately from 0.12 to 0.49%, reflecting different experimental strategies, including phosphorus-adequate diets and diets deliberately reduced in this mineral to evaluate the efficacy of enzyme supplementation.

The variables analyzed in the included studies primarily encompassed eggshell quality parameters, including shell thicknes, SBS, and shell percentage, as well as egg SG in some experiments. Together, these variables allowed a comprehensive assessment of the effects of phytase supplementation on mineral utilization and eggshell structural quality in laying hens.

### Meta-analysis results

#### Shell weight percentage

**Fig. 2:**
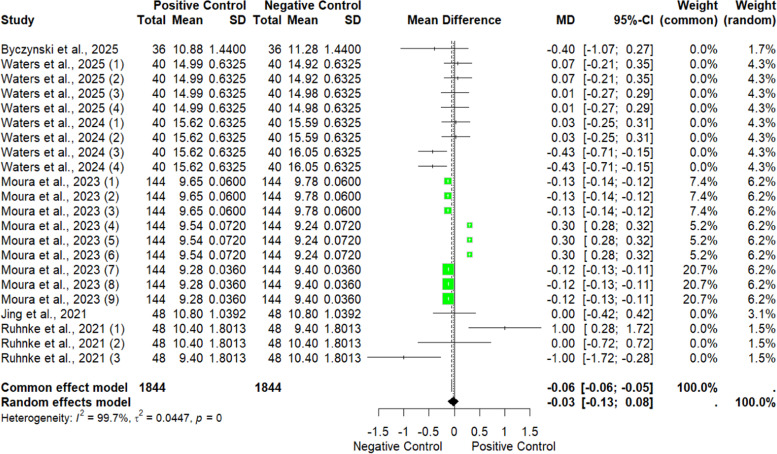
Forest plot of the meta-analysis comparing the shell percentage of eggs from laying hens fed positive control diets (PC; diets with adequate available phosphorus levels) and negative control diets (NC; diets with reduced available phosphorus levels). "Total represents the sample size (n) of each experimental group included in the meta-analysis".

The meta-analysis of SWP revealed high heterogeneity among studies (I² > 98%), requiring interpretation based on random-effects models. In the comparison between the PC and the NC, no significant difference was observed (MD = −0.03; 95% CI: −0.13 to 0.08; p = 0.633), indicating that dietary phosphorus restriction did not markedly reduce the proportional contribution of the shell to the egg (Fig. 2).

**Fig. 3:**
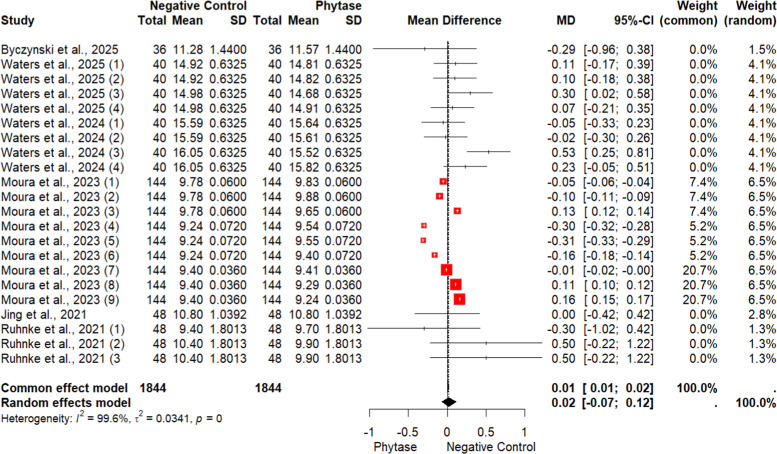
Forest plot of the meta-analysis evaluating the effect of phytase supplementation on egg shell percentage in laying hens, comparing negative control diets (NC; reduced available phosphorus) with phytase-supplemented diets. "Total represents the sample size (n) of each experimental group included in the meta-analysis".

Similarly, phytase supplementation compared with the NC (NC vs. Phytase) did not result in a significant overall effect on this variable (MD = 0.02; 95% CI: −0.07 to 0.12; p = 0.631), as illustrated in Fig. 3.

**Fig. 4:**
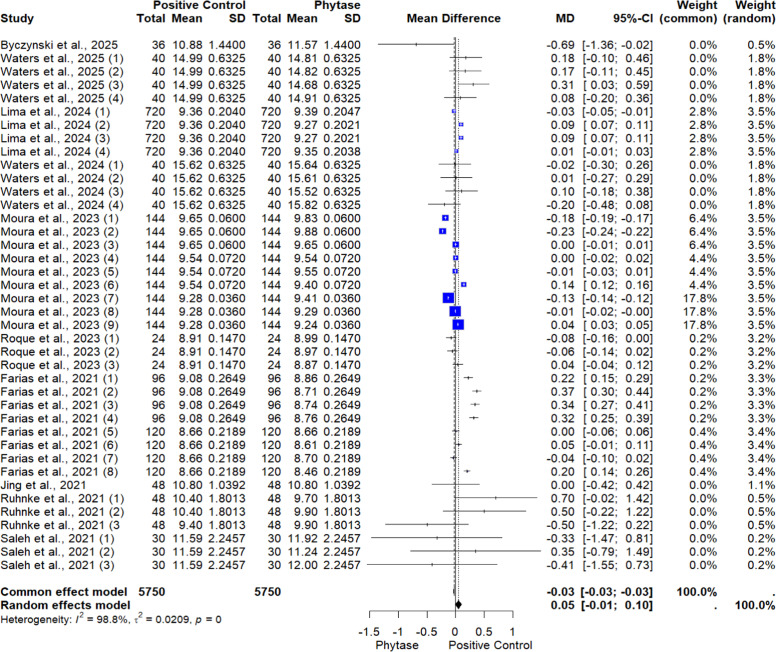
Forest plot of the meta-analysis comparing the shell percentage of eggs from laying hens fed positive control diets (PC; adequate available phosphorus levels) and phytase-supplemented diets. "Total represents the sample size (n) of each experimental group included in the meta-analysis".

Finally, in the comparison between the PC and the phytase-supplemented groups (PC vs. Phytase), a trend toward a higher SWP was observed in the phytase groups; however, this difference did not reach statistical significance (MD = 0.05; 95% CI: −0.01 to 0.10; p = 0.081), as shown in Fig. 4.

Detailed subgroup analysis results are summarized in Table 2. Stratification according to the difference in available phosphorus (P-diff) demonstrated that the intensity of the nutritional challenge did not significantly influence the response. In the contrast between the PC and the NC, no differences were observed between the P-diff subgroups (p = 0.527). Similarly, when comparing the PC group with phytase-supplemented hens, the analysis suggested that the efficacy of phytase in maintaining SWP was consistent regardless of the magnitude of the initial phosphorus deficiency (p = 0.110).

**Table 2:** Effects of treatment contrasts on egg shell weight percentage in laying hens, including mean difference, 95% confidence interval, and heterogeneity measures.

Contrast	Factor	Subgroup	Mean Difference (%)		Heterogeneity		
			k	IV. Random. 95% CI	Tau²	p-value	I²
PC x NC	P Diff	≤0.2%	512	−0.06 [−0.23; 0.10]	0.049	0.001	65.2
		>0.2%	1332	0.00 [−0.13; 0.14]	0.045	0.000	99.9
NC x Phytase	Phytase type	Bacterial	1664	0.02 [−0.08; 0.11]	0.034	0.000	99.7
		Fungal	180	0.09 [−0.36; 0.54]	0.081	0.182	38.3
	Phytase level	≤600 FTU/kg	1024	−0.00 [−0.15; 0.15]	0.051	0.000	99.7
		600-1000 FTU/kg	480	0.04 [−0.13; 0.21]	0.026	<0.001	99.7
		>1000 FTU/kg	340	0.09 [−0.04; 0.22]	<0.001	0.486	0.0
PC x Phytase	P Diff	≤0.2%	4202	0.08 [0.02; 0.14]	0.016	<0.001	91.0
		>0.2%	1548	−0.01 [−0.11; 0.09]	0.026	0.000	99.6
	Phytase type	Bacterial	5078	0.02 [−0.03; 0.07]	0.015	0.000	99.0
		Fungal	672	0.14 [−0.05; 0.34]	0.055	<0.001	91.4
	Phytase level	≤600 FTU/kg	2224	0.00 [−0.07; 0.08]	0.021	<0.001	98.9
		600-1000 FTU/kg	1656	0.13 [0.05; 0.21]	0.016	<0.001	97.2
		>1000 FTU/kg	1870	0.04 [−0.03; 0.10]	0.003	<0.001	73.2

PC: positive control (diet with adequate available phosphorus levels); NC: negative control (diet with reduced available phosphorus levels); Phytase: negative control diet supplemented with phytase; P Diff: difference in available phosphorus level between treatments; k: number of observations; 95% CI: 95% confidence interval; Tau²: between-study variance; I²: percentage of total variability attributable to between-study heterogeneity.

Regarding phytase type, enzyme origin (fungal or bacterial) did not result in statistically significant differences. Both in the comparison between the NC and Phytase (p = 0.753) and in the contrast between the PC and Phytase (p = 0.248), results were comparable across the enzymatic sources evaluated.

Stratification by phytase dose also revealed no significant differences in response when compared with the NC (p = 0.629), indicating that restoration of SP was not dependent on the inclusion range (≤600, 600–1,000, or >1,000 FTU/kg). However, in the comparison between the PC and phytase-supplemented hens, a trend toward variation among dosage levels was observed (p = 0.067), suggesting that the ability of phytase to match the SWP observed in the PC may be influenced by the dose applied, although the heterogeneity of the data warrants cautious interpretation.

#### Shell thickness

**Fig. 5:**
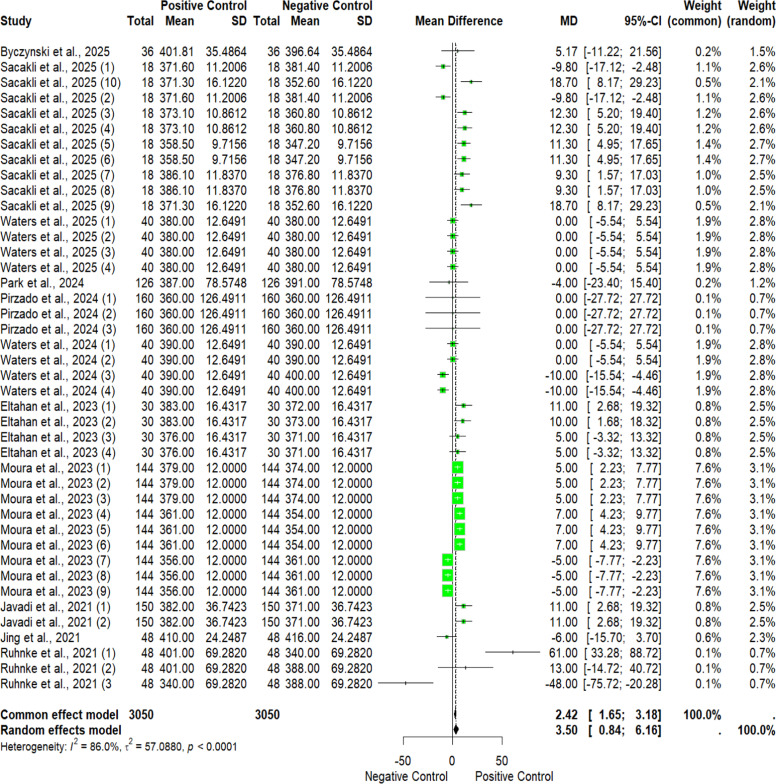
Forest plot of the meta-analysis comparing eggshell thickness in laying hens fed positive control diets (PC; diets with adequate available phosphorus levels) and negative control diets (NC; diets with reduced available phosphorus levels). "Total represents the sample size (n) of each experimental group included in the meta-analysis".

For ST, the overall results indicate that the nutritional challenge imposed by the NC significantly reduced shell quality, whereas phytase supplementation exerted restorative effects. In the comparison between the PC and the NC, a significant reduction in ST was observed in the NC group (MD = 3.50; 95% CI: 0.84 to 6.16; p = 0.010), as illustrated in Fig. 5.

**Fig. 6:**
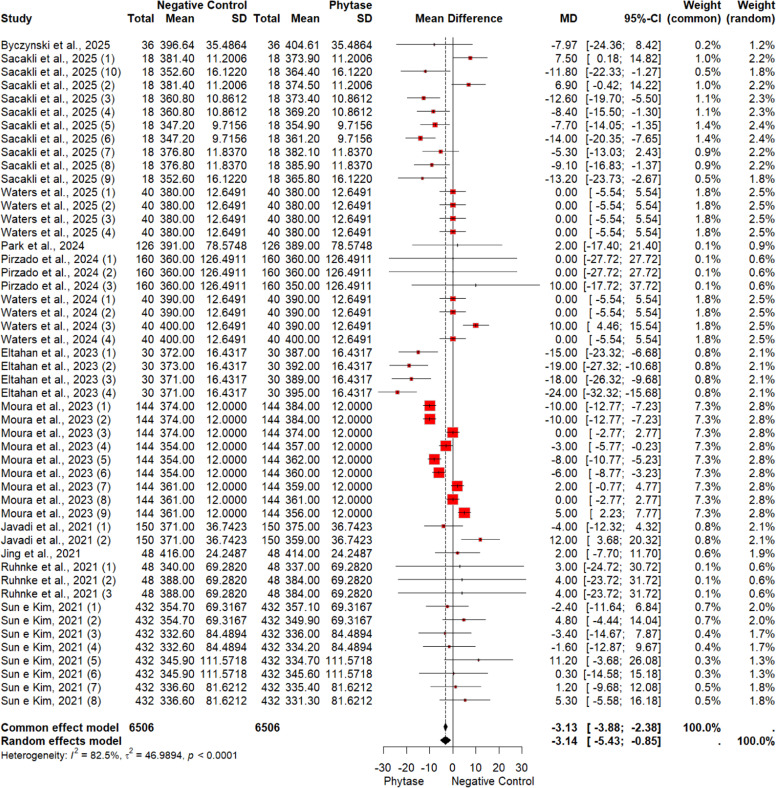
Forest plot of the meta-analysis evaluating the effect of phytase supplementation on eggshell thickness in laying hens, comparing negative control diets (NC; reduced available phosphorus) with phytase-supplemented diets. "Total represents the sample size (n) of each experimental group included in the meta-analysis".

Phytase supplementation compared with the NC (NC vs. Phytase) resulted in a significant increase in ST (MD = −3.14; 95% CI: −5.43 to −0.85; p = 0.007), demonstrating the effectiveness of the enzyme in mitigating the adverse effects of phosphorus restriction (Fig. 6).

**Fig. 7:**
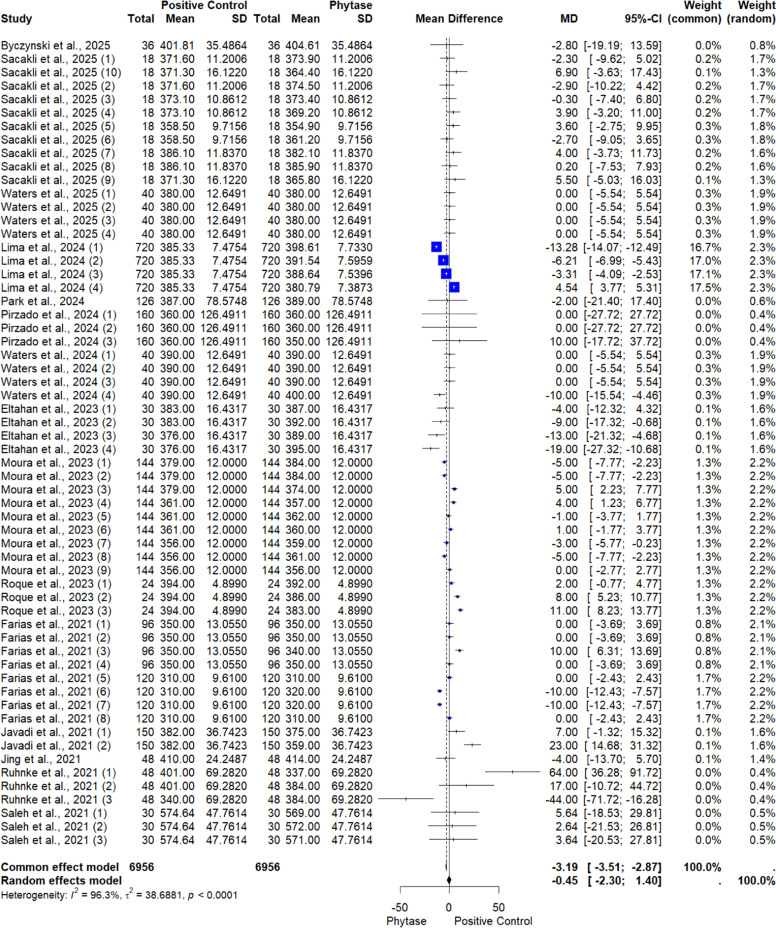
Forest plot of the meta-analysis comparing eggshell thickness in laying hens fed positive control diets (PC; adequate available phosphorus levels) and phytase-supplemented diets. "Total represents the sample size (n) of each experimental group included in the meta-analysis".

Finally, in the comparison between the PC and the phytase-supplemented groups (PC vs. Phytase), no significant difference was observed under the random-effects model (MD = −0.45; 95% CI: −2.30 to 1.40; p = 0.632), indicating that phytase was able to restore ST to levels comparable to those observed in the PC group (Fig. 7).

Detailed subgroup analysis results are summarized in Table 3. Stratification according to the difference in available phosphorus (P-diff) revealed that the impact of nutritional restriction on ST reduction depended on the magnitude of the imposed deficiency (p = 0.001 for the PC vs. NC contrast). In contrast, when comparing the PC with phytase-supplemented hens, no significant differences were observed among P-diff levels (p = 0.629), indicating that phytase supplementation consistently restored ST regardless of the severity of the initial P restriction.

**Table 3:** Effects of treatment contrasts on eggshell thickness in laying hens, including mean difference, 95% confidence interval, and heterogeneity measures.

Contrast	Factor	Subgroup	Mean Difference (µm)		Heterogeneity		
			k	IV. Random. 95% CI	Tau²	p-value	I²
PC x NC	P Diff	≤0.2%	1118	−2.17 [−5.17; 1.41]	24.691	<0.001	70.6
		>0.2%	1932	5.75 [2.87; 8.63]	44.592	<0.001	88.2
NC x Phytase	Phytase type	Bacterial	5426	−2.16 [−4.22; −0.09]	28.286	<0.001	82.5
		Fungal	1080	−7.02 [−14.44; 0.41]	114.61	<0.001	78.2
	Phytase level	≤600 FTU/kg	1640	−3.95 [−6.84; −1.06]	37.274	<0.001	84.7
		600-1000 FTU/kg	910	−6.47 [−14.12; 1.18]	130.84	<0.001	92.0
		>1000 FTU/kg	3956	0.46 [−1.76; 2.67]	0.000	0.984	0.0
PC x Phytase	P Diff	≤0.2%	4808	0.02 [−2.67; 2.71]	41.081	<0.001	97.8
		>0.2%	2148	−0.90 [−3.49; 1.69]	38.200	<0.001	85.3
	Phytase type	Bacterial	5384	−0.57 [−2.23; 1.09]	22.491	<0.001	97.2
		Fungal	1572	0.83 [−6.74; 8.40]	217.19	<0.001	90.2
	Phytase level	≤600 FTU/kg	2840	−0.30 [−2.40; 1.79]	25.342	<0.001	95.9
		600-1000 FTU/kg	2086	−1.47 [−6.15; 3.21]	79.247	<0.001	95.1
		>1000 FTU/kg	2030	1.62 [−6.03; 9.27]	146.90	<0.001	94.7

PC: positive control (diet with adequate available phosphorus levels); NC: negative control (diet with reduced available phosphorus levels); Phytase: negative control diet supplemented with phytase; P Diff: difference in available phosphorus level between treatments; k: number of observations; 95% CI: 95% confidence interval; Tau²: between-study variance; I²: percentage of total variability attributable to between-study heterogeneity.

Regarding phytase type, enzyme origin (fungal or bacterial) did not result in significant differences. Both in the comparison between the NC and Phytase (p = 0.216) and in the contrast between the PC and Phytase (p = 0.723), the efficacy of supplementation remained consistent across enzymatic sources.

Stratified analysis by phytase dose revealed that variation in enzyme inclusion influenced the response in the contrast between the NC and phytase-supplemented hens (p = 0.025), suggesting that restoration of ST depends on the supplementation level. However, when comparing the PC with the supplemented groups, no significant differences were observed among dosage levels (p = 0.789), reinforcing that phytase supplementation, regardless of dose, restored ST to values comparable to those of the reference group.

#### Shell breaking strength

**Fig. 8:**
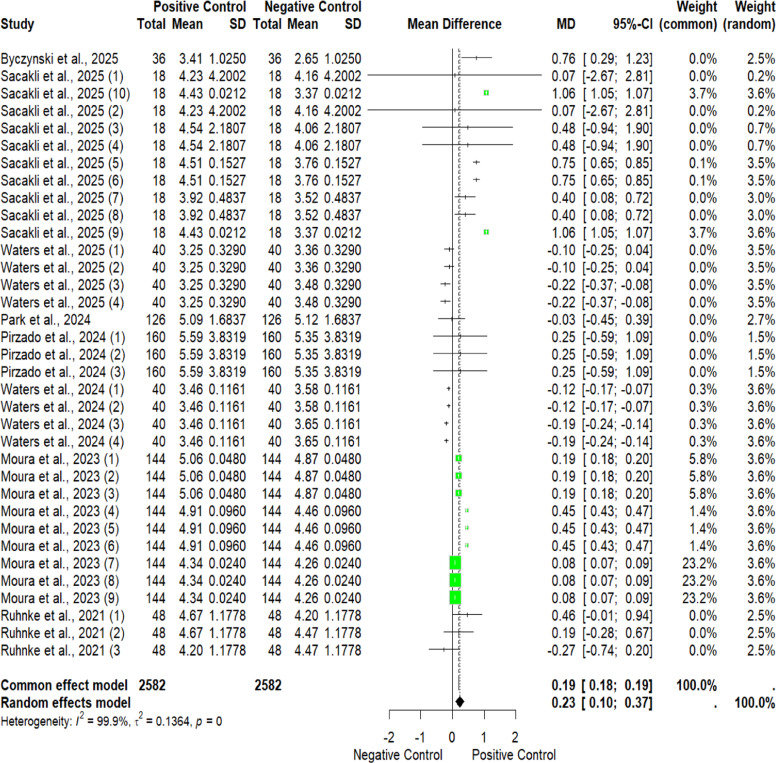
Forest plot of the meta-analysis comparing eggshell breaking strength in laying hens fed positive control diets (PC; diets with adequate available phosphorus levels) and negative control diets (NC; diets with reduced available phosphorus levels). "Total represents the sample size (n) of each experimental group included in the meta-analysis".

The meta-analytical evaluation of SBS revealed a consistent pattern between the nutritional challenge and the response to enzymatic supplementation. In the comparison between the Positive Control (PC) and the Negative Control (NC), a significant reduction in shell strength was observed in hens subjected to phosphorus restriction (MD = 0.23 kgf; 95% CI: 0.10 to 0.37; p = 0.001), as illustrated in Fig. 8.

**Fig. 9:**
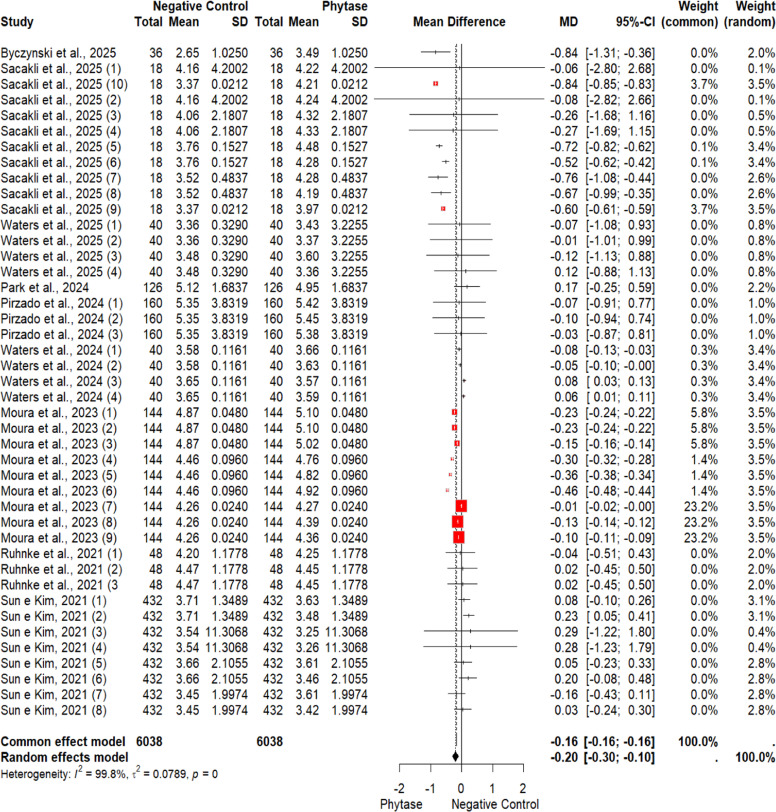
Forest plot of the meta-analysis evaluating the effect of phytase supplementation on eggshell breaking strength in laying hens, comparing negative control diets (NC; reduced available phosphorus) with phytase-supplemented diets. "Total represents the sample size (n) of each experimental group included in the meta-analysis".

When assessing the effect of phytase relative to the negative control (NC vs. Phytase), a significant increase in shell strength was observed (MD = −0.20 kgf; 95% CI: −0.30 to −0.10; p = 0.001), demonstrating the enzyme’s ability to mitigate the adverse effects of phosphorus deficiency (Fig. 9).

**Fig. 10:**
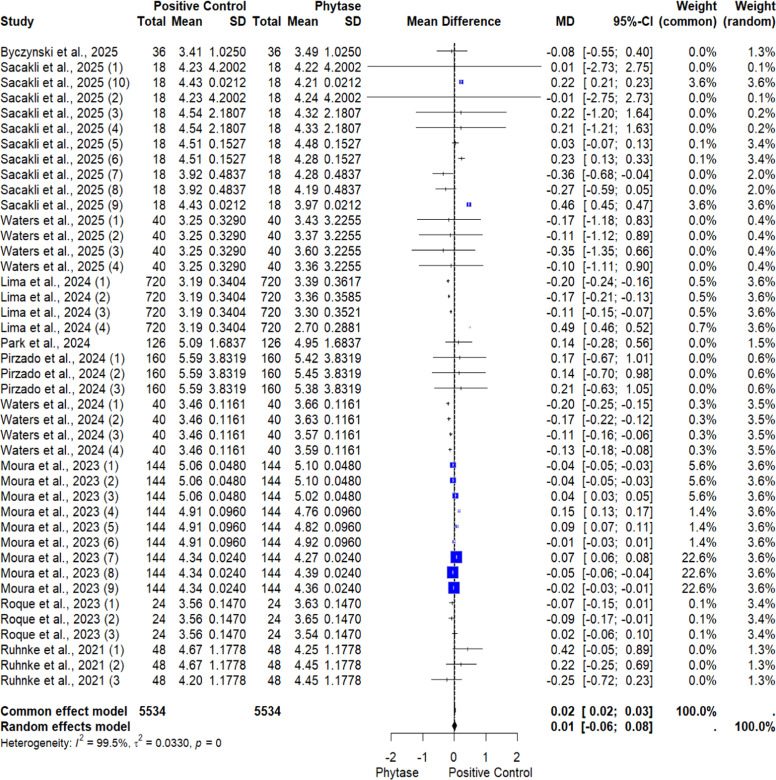
Forest plot of the meta-analysis comparing eggshell breaking strength in laying hens fed positive control diets (PC; adequate available phosphorus levels) and phytase-supplemented diets. "Total represents the sample size (n) of each experimental group included in the meta-analysis".

Finally, in the comparison between the Positive Control and phytase-supplemented hens (Fig. 10), no statistically significant differences were detected (MD = 0.01 kgf; 95% CI: −0.06 to 0.08; p = 0.815). These findings indicate that phytase supplementation effectively restores SBS to levels comparable to those observed in the positive control group.

Detailed subgroup analysis results are summarized in Table 4. Stratification according to the difference in available phosphorus (P-diff) demonstrated that the magnitude of nutritional restriction influences shell integrity. In the contrast between the Positive Control and the Negative Control, a highly significant difference was observed among P-diff levels (p < 0.001), indicating that shell resistance to nutritional challenge varies according to the severity of phosphorus deficiency. In contrast, when comparing the Positive Control with phytase-supplemented hens, no significant differences were detected among P-diff categories (p = 0.156), reinforcing that phytase restores shell strength regardless of the severity of the initial phosphorus restriction.

**Table 4:** Effects of treatment contrasts on eggshell breaking strength in laying hens, including mean difference, 95% confidence interval, and heterogeneity measures.

Contrast	Factor	Subgroup	Mean Difference (kgf)		Heterogeneity		
			k	IV. Random. 95% CI	Tau²	p-value	I²
PC x NC	P Diff	≤0.2%	1070	−0.15 [−0.19; −0.12]	0.001	0.071	37.5
		>0.2%	1512	0.45 [0.29; 0.61]	0.107	0.000	99.9
NC x Phytase	Phytase type	Bacterial	5378	−0.20 [−0.31; −0.09]	0.082	0.000	99.8
		Fungal	660	−0.17 [−0.46; 0.12]	0.060	0.152	36.2
	Phytase level	≤600 FTU/kg	1490	−0.33 [−0.47; −0.19]	0.083	0.000	99.9
		600-1000 FTU/kg	592	−0.23 [−0.44; −0.02]	0.035	<0.001	99.7
		>1000 FTU/kg	3956	0.02 [−0.06; 0.11]	0.089	0.011	49.7
PC x Phytase	P Diff	≤0.2%	4022	−0.04 [−0.14; 0.06]	0.036	<0.001	98.3
		>0.2%	1512	0.06 [−0.03; 0.14]	0.025	0.000	99.7
	Phytase type	Bacterial	4874	0.00 [−0.07; 0.07]	0.034	0.000	99.5
		Fungal	660	0.10 [−0.12; 0.32]	0.007	0.578	0.0
	Phytase level	≤600 FTU/kg	2258	0.01 [−0.08; 0.09]	0.033	0.000	99.6
		600-1000 FTU/kg	1336	−0.03 [−0.10; 0.04]	0.007	<0.001	97.0
		>1000 FTU/kg	1940	0.04 [−0.15; 0.23]	0.063	<0.001	98.9

PC: positive control (diet with adequate available phosphorus levels); NC: negative control (diet with reduced available phosphorus levels); Phytase: negative control diet supplemented with phytase; P Diff: difference in available phosphorus level between treatments; k: number of observations; 95% CI: 95% confidence interval; Tau²: between-study variance; I²: percentage of total variability attributable to between-study heterogeneity.

Regarding phytase type, enzyme source (fungal or bacterial) did not affect supplementation efficacy. Subgroup analysis revealed no significant differences between enzyme sources either in the comparison with the negative control (p = 0.834) or relative to the positive control (p = 0.409).

Stratification by phytase dose revealed that, in the comparison between the Negative Control and phytase-supplemented hens, significant differences were observed among dosage subgroups (p < 0.001), suggesting that restoration of shell strength in phosphorus-deficient diets is dose-dependent. However, when comparing the Positive Control with the supplemented groups, no significant differences were found among dosage ranges (p = 0.724), confirming that phytase supplementation, regardless of dose (≤600, 600–1,000, or >1,000 FTU/kg), maintains shell strength at levels equivalent to those of the positive control group.

#### Specific gravity

The analysis of SG yielded limited results due to the small number of studies available for certain comparisons. In the contrast between the PC and the NC, the available data did not indicate a statistically significant difference (p = 0.157). Similarly, in the comparison between the NC and phytase-supplemented groups, the presence of only one study precluded a formal meta-analysis.

**Fig. 11:**
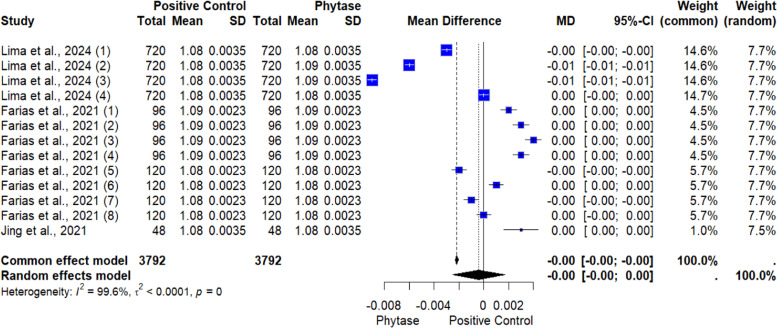
Forest plot of the meta-analysis comparing egg specific gravity in laying hens fed positive control diets (PC; diets with adequate available phosphorus levels) and negative control diets (NC; diets with reduced available phosphorus levels). "Total represents the sample size (n) of each experimental group included in the meta-analysis".

However, when comparing the PC with phytase-supplemented hens, the random-effects model showed no significant difference (MD = 0.00; p = 0.709), indicating that phytase inclusion maintains SG at levels equivalent to those observed in nutritionally adequate diets (Fig. 11).

Detailed subgroup analysis results for this contrast are summarized in Table 5. Due to the limited availability of data, subgroup analyses were only possible for the comparison between the Positive Control and phytase-supplemented groups. Stratification according to the magnitude of available phosphorus reduction (P-diff) revealed no significant differences among subgroups (p = 0.072), suggesting that variation in mineral deficiency does not compromise the ability of phytase to maintain SG.

**Table 5:** Effects of treatment contrasts on egg specific gravity in laying hens, including mean difference, 95% confidence interval, and heterogeneity measures.

Contrast	Factor	Subgroup	Mean Difference (g/cm³)		Heterogeneity		
			k	IV. Random. 95% CI	Tau²	p-value	I²
PC x Phytase	P Diff	≤0.2%	3576	−0.00 [−0.00; 0.00]	<0.001	0.000	99.6
		>0.2%	216	0.00 [0.00; 0.00]	<0.001	<0.001	95.1
	Phytase type	Bacterial	3360	−0.00 [−0.00; 0.00]	<0.001	0.000	99.7
		Fungal	432	0.00 [−0.00; 0.00]	<0.001	<0.001	98.3
	Phytase level	≤600 FTU/kg	1152	−0.00 [−0.00; 0.00]	<0.001	<0.001	99.1
		600-1000 FTU/kg	1200	0.00 [−0.00; 0.00]	<0.001	<0.001	99.6
		>1000 FTU/kg	1440	−0.00 [−0.01; 0.00]	<0.001	<0.001	99.9

PC: positive control (diet with adequate available phosphorus levels); NC: negative control (diet with reduced available phosphorus levels); Phytase: negative control diet supplemented with phytase; P Diff: difference in available phosphorus level between treatments; k: number of observations; 95% CI: 95% confidence interval; Tau²: between-study variance; I²: percentage of total variability attributable to between-study heterogeneity.

Regarding phytase type, enzyme origin (bacterial or fungal) did not affect comparative performance, with the random-effects model indicating similar results across subgroups (p = 0.136). Likewise, variation in phytase dose (≤600, 600–1,000, or >1,000 FTU/kg) did not influence enzyme efficacy (p = 0.5498), demonstrating that SG remains stable and equivalent to that of the positive control group regardless of the supplementation level evaluated.

## Discussion

The results of the present meta-analysis demonstrate that phytase supplementation in diets for laying hens plays an important role in maintaining eggshell quality, particularly under conditions of available phosphorus restriction. Overall, structural shell parameters, such as ST and SBS, were more sensitive to nutritional changes than SWP and SG, suggesting that different variables respond distinctly to alterations in mineral metabolism in laying hens (Shet et al., 2018; Taylor et al., 2018; Sundu et al., 2023). Recent evidence in older laying hens (68–78 weeks of age) further supports that severe phosphorus deficiency (0.12% available phosphorus) significantly impairs shell strength, an effect that can be mitigated by supplementation with 600 FTU/kg of phytase (Bello et al., 2020; Sacakli et al., 2025). In addition, the use of next-generation bacterial phytases in diets devoid of inorganic phosphorus has been shown to restore SBS to levels comparable to those observed in nutritionally adequate diets (Sacakli et al., 2025).

The absence of significant differences in SWP between the positive and negative control groups indicates that phosphorus restriction does not necessarily alter the relative proportion of shell in relation to total egg weight (Pongmanee et al., 2020). This finding may be explained by the fact that SWP is a variable dependent on egg weight and the relative deposition of calcium carbonate during shell formation in the uterus (Jin et al., 2024). Even under mineral restriction, hens may maintain the structural proportion of the shell through compensatory physiological mechanisms, primarily via mobilization of mineral reserves from medullary bone. This specialized bone tissue, present in laying hens, acts as a dynamic reservoir of calcium and phosphorus, allowing the bird to temporarily sustain shell mineralization even when dietary intake of these minerals is limited (Alfonso-Carrillo et al., 2021). Long-term studies (30–70 weeks) confirm that laying hens can physiologically adapt to moderate mineral reductions, maintaining shell quality at the expense of bone mineralization until these reserves are no longer sufficient to support production (Bello and Korver, 2019; Bello et al., 2020). Conversely, phytase superdosing (above 2,500 FTU/kg) has been shown to increase shell mass and density, possibly due to the additional release of calcium from phytate complexes (Byczyński et al., 2025). On the other hand, in hens at the end of the laying cycle (70–86 weeks), supplementation with 300 FTU/kg in reduced-phosphorus diets improved egg production by 2.68%, although ST remained higher in the control group receiving adequate nutrient levels (Roque et al., 2023).

In contrast, ST was significantly reduced in the nutritionally restricted group, demonstrating that available phosphorus deficiency directly compromises shell mineralization (Pongmanee et al., 2020). Phosphorus plays a central role in calcium metabolism and mineral homeostasis, participating in cellular processes related to intestinal transport, bone deposition, and energy metabolism (Liao et al., 2022). Deficiency of this nutrient may impair intestinal calcium absorption and alter acid–base balance during shell calcification, resulting in insufficient calcium carbonate deposition during egg formation. In this context, phytase acts by hydrolyzing phytic acid present in plant-based feed ingredients, releasing previously unavailable phosphorus and increasing its bioavailability for avian metabolism (Ribeiro et al., 2024a). In quail subjected to heat stress (36°C), supplementation with 1,500 FTU/kg of phytase not only increased ST but also stimulated the expression of the calcium transporter calbindin-D28k in the uterus, facilitating calcification under high-temperature conditions (Ribeiro et al., 2024b; Silva et al., 2026). This regulatory role also extends to the hormonal profile of older hens (73–80 weeks), in which the enzyme increases plasma levels of FSH and estradiol, thereby supporting the persistence of external egg quality (Eltahan et al., 2023). However, when dietary calcium restriction is severe (20 g/kg), phytase may not exert a significant main effect on shell parameters, indicating that calcium availability remains the primary limiting factor (Ruhnke et al., 2021). Current research suggests that supplementation levels around 1,217 FTU/kg are optimal for maximizing ST, while higher doses combined with specific limestone particle sizes enhance SBS in hens at the end of the production cycle (Lima et al., 2024; Waters et al., 2025).

The restorative effect of phytase observed on ST reinforces the importance of this enzyme in improving phosphorus availability and, consequently, shell mineralization efficiency. In Japanese quail exposed to severe heat stress (36°C), superdosing with 1,500 FTU/kg of phytase proved crucial in mitigating reductions in ST, an effect mediated by increased uterine expression of calbindin-D28k (Ribeiro et al., 2024b; Silva et al., 2026). Similarly, in older laying hens (73–80 weeks), supplementation with 1,000 FTU/kg of phytase not only improved ST but also increased plasma concentrations of albumin, calcium, and phosphorus, enabling dietary reductions without compromising external egg quality (Eltahan et al., 2023). Phytate hydrolysis not only releases phosphorus but also reduces the formation of insoluble complexes between phytate and divalent minerals such as calcium, zinc, and iron (Saleh et al., 2021; Ribeiro et al., 2024a). This effect enhances the availability of these minerals for intestinal absorption and metabolic utilization, thereby contributing to physiological processes associated with shell formation and skeletal integrity (Ribeiro et al., 2024a).

Similar results were observed for SBS, a variable directly associated with structural integrity and the crystalline organization of calcium carbonate. The reduction in shell strength in the negative control group indicates that phosphorus deficiency compromises not only the quantity but also the quality of the mineral matrix deposited during eggshell calcification (Hervo et al., 2023). Studies in Bovans Brown laying hens have shown that supplementation with phytase B (6,575 AcPU/kg) can significantly increase shell mass, density, and SBS, reaching values even higher than those observed in diets containing standard mineral levels (Byczyński et al., 2025). Furthermore, the efficacy of phytase on shell strength appears to interact with limestone particle size, as the combination of 1,500 FTU/kg with a mixture of 40% fine and 60% coarse limestone maximized SBS in late-cycle hens (Waters et al., 2025). Shell strength is closely related to the organization of calcite crystals and the presence of an organic protein matrix that serves as a scaffold for mineral deposition. Alterations in mineral metabolism may interfere with the proper formation of this structure, resulting in more fragile shells and increased susceptibility to breakage (Zhao et al., 2024).

Phytase supplementation proved effective in restoring SBS to levels comparable to those of the positive control, highlighting its capacity to improve dietary phosphorus utilization and optimize mineral metabolism in laying hens (Sun and Kim, 2021). However, the response may vary; while some studies using hybrid bacterial phytases (300–600 FTU/kg) reported complete restoration of shell strength and tibial ash content (Jlali et al., 2023; Sacakli et al., 2025), others observed that under conditions of extreme calcium deficiency, the effect of the enzyme on shell quality may be limited, as systemic mineral homeostasis takes priority (Ruhnke et al., 2021). This restorative effect is associated not only with increased phosphorus availability but also with improved amino acid and energy digestibility, since phytate also acts as an antinutritional factor that can reduce the digestive efficiency of several nutrients. Additionally, in older hens, phytase supports laying persistence by increasing levels of reproductive hormones such as FSH and estradiol, suggesting an endocrine benefit that complements enhanced nutritional efficiency (Eltahan et al., 2023). Thus, phytase contributes to improved overall metabolic efficiency, which is positively reflected in eggshell formation (Selle et al., 2023).

Subgroup analysis demonstrated that the magnitude of phosphorus restriction significantly influences certain responses, particularly ST and SBS (Pongmanee et al., 2020; Sun and Kim, 2021; Hervo et al., 2023). These findings suggest that the impact of mineral deficiency depends on the severity of the nutritional challenge imposed. In studies involving older laying hens (68–78 weeks), supplementation with 600 FTU/kg was able to fully restore bone quality under moderate restriction but resulted only in partial recovery under severe restriction, indicating that drastic reductions may exceed the phosphorus-releasing potential of standard phytase doses (Bello et al., 2020). Nevertheless, when phytase is supplemented, responses tend to become more uniform across different levels of restriction, indicating that the enzyme can at least partially compensate for reductions in dietary available phosphorus. This adaptive capacity is further supported by physiological mechanisms, such as increased renal expression of the NaPi-IIa transporter, which acts in conjunction with phytase to maintain mineral homeostasis under conditions of limited dietary phosphorus supply (Ren et al., 2023).

Another relevant finding of this meta-analysis was the absence of significant differences between bacterial and fungal phytases (Shet et al., 2018; Saleh et al., 2021). This suggests that, regardless of the microbial origin of the enzyme, its capacity to hydrolyze phytate and release phosphorus is sufficient to promote similar effects on eggshell quality parameters. Although some individual studies have reported superior egg production with bacterial phytase derived from Escherichia coli compared with fungal phytase, other studies using superdoses (5,000 FTU/kg) have confirmed that both bacterial and fungal sources (*Aspergillus niger* and *Trichoderma reesei*) are equally effective in completely replacing inorganic phosphorus supplementation (Farias et al., 2021; Saleh et al., 2021). Although there are structural and kinetic differences among commercially available phytases, the results indicate that, under the conditions evaluated, these differences do not translate into distinct productive responses (Saleh et al., 2021).

However, evidence from broader meta-analyses suggests that enzyme origin may influence phosphorus release potential. Rostagno et al. (2024) conducted a systematic review and meta-analysis involving 106 studies to estimate the equivalency of available phosphorus and standardized digestible phosphorus released by phytase supplementation in broiler diets. The authors observed a progressive increase in phosphorus equivalency as enzyme inclusion levels increased (250 to 1,500 FTU/kg), indicating a positive response to phytase supplementation. Furthermore, the analysis showed that bacterial phytases exhibited, on average, a greater phosphorus-releasing capacity than fungal phytases at equivalent doses, demonstrating that enzyme origin can affect phytate hydrolysis efficiency. Recent studies using hybrid bacterial phytase variants (300–600 FTU/kg) have shown that these newer generations can restore eggshell breaking strength and bone mineralization to levels comparable to those achieved with nutritionally adequate diets, suggesting advances in catalytic efficiency (Jlali et al., 2023; Sacakli et al., 2025).

In addition to enzyme origin, phytase inclusion level may also influence the magnitude of the productive response. Regarding phytase dosage, the analysis indicated that different inclusion levels can affect the magnitude of response for some variables, particularly when compared with the negative control. This finding suggests the existence of a dose-response relationship associated with the enzyme’s capacity to release phosphorus from dietary phytate. While doses of 1,000 FTU/kg appear necessary for long-term benefits in calcium and phosphorus digestibility, the use of superdoses (up to 2,000 FTU/kg) has resulted in linear improvements in laying rate, egg mass, and feed conversion, in some cases even surpassing standard diets (Javadi et al., 2021; Pirzado et al., 2024). However, when compared with the positive control, different inclusion levels produced similar responses, indicating that moderate doses may already be sufficient to restore the mineral availability required for proper eggshell formation (Farias et al., 2021; Pirzado et al., 2024; Ribeiro et al., 2025). Nevertheless, to optimize variables such as eggshell thickness and intestinal morphology in birds at the end of the production cycle, phytase levels between 1,217 and 1,500 FTU/kg have been recommended as the optimal range for productive and physiological efficiency (Lima et al., 2024; Silva et al., 2026).

Finally, the results related to SG were limited by the small number of available studies. Even so, the absence of significant differences among treatment groups suggests that this variable may be less sensitive to nutritional changes, or that its response depends on other factors such as bird age, egg weight, and management conditions (Lima et al., 2011; Silva et al., 2012). Several studies support this low sensitivity, reporting that mineral reduction and phytase supplementation often do not alter SG, even when other eggshell parameters are affected (Bello et al., 2019; Jing et al., 2021; Farias et al., 2021). SG is widely used as an indirect indicator of eggshell quality, but its ability to detect subtle changes in mineralization may be lower than that of more direct measurements, such as ST and SBS.

Under conditions of severe mineral restriction, however, SG may decline significantly, as observed in older laying hens (70 to 78 weeks of age) fed diets deficient in calcium and phosphorus (Bello et al., 2020). In such cases, supplementation with 600 FTU/kg of phytase was effective in restoring this parameter to levels comparable to the positive control (Bello et al., 2020). On the other hand, studies involving phytase superdosing indicate that higher inclusion levels (estimated at 1,750 FTU/kg) may promote linear and quadratic improvements in SG, suggesting that doses above conventional recommendations can optimize mineral deposition more robustly (Lima et al., 2024).

In Japanese quail, the results also support the stability of this parameter. Even under variations in available phosphorus levels and supplementation with 500 FTU/kg of phytase, no changes in SG were observed over a 21-day evaluation period (Rossetto et al., 2019). Likewise, under heat stress conditions (36°C) and the use of superdoses up to 3,000 FTU/kg, SG remained unchanged, whereas eggshell thickness responded positively to supplementation with 1,500 FTU/kg (Ribeiro et al., 2024b; Silva et al., 2026). This discrepancy reinforces that SG may not adequately reflect phytase efficiency in mobilizing calcium to the shell gland, a process better demonstrated by ST measurements and by the expression of transport proteins such as calbindin-D28k (Ribeiro et al., 2024a, 2024b; Silva et al., 2026). Therefore, although values equal to or greater than 1.080 g/cm³ indicate good eggshell quality, the absence of a statistical response among treatments highlights the need for complementary structural assessments to validate enzymatic efficacy (Farias et al., 2021).

Taken together, the results of this meta-analysis indicate that phytase supplementation is an effective nutritional strategy for improving dietary phosphorus utilization and mitigating the negative effects of phosphorus-deficient diets on eggshell quality. The enzyme contributes to maintaining eggshell structural integrity by enhancing the bioavailability of essential minerals and optimizing mineral metabolism in laying hens, thereby representing an important tool for more efficient and sustainable egg production systems.

## Conclusion

Phytase supplementation in diets for laying hens is effective in mitigating the adverse effects of phosphorus restriction on eggshell quality. The enzyme primarily restores ST and SBS to levels comparable to those of the positive control, whereas SWP and SG appear less sensitive to nutritional alterations. Furthermore, neither phytase origin nor the range of inclusion levels evaluated significantly affected the overall responses. Therefore, phytase represents an efficient strategy for optimizing phosphorus utilization and maintaining eggshell quality.

## Author contribution

Adiel Vieira de Lima: Conceived and designed the study, performed the literature search, screened and selected eligible studies, conducted the data analysis, interpreted the results, and drafted the manuscript. Matheus Ramalho de Lima: Contributed to study design, participated in literature screening and data analysis, and contributed to interpretation of the results and manuscript development. Amanda Itoh de Medeiros: Contributed to literature search and study selection, and participated in data interpretation. José Henrique Stringhini: Contributed to literature search and study selection, and participated in data interpretation. Thácyla Beatriz Duarte Correia: Contributed to literature search and study selection, and participated in data interpretation. Apolônio Gomes Ribeiro: Contributed to study design, participated in data analysis and interpretation, and contributed to manuscript development. Ilaiane Barbosa Matias Barros: Participated in data analysis and contributed to interpretation of the results. Luayne Morais Correa: Participated in data analysis and contributed to interpretation of the results. Jamilly Lima Ferreira Oliveira: Participated in data analysis and contributed to interpretation of the results. Janete Gouveia de Souza: Contributed to literature organization, participated in data analysis, and contributed to interpretation of the results. Ricardo Romão Guerra: Contributed to literature organization, participated in data analysis, and contributed to interpretation of the results. Lucas Rannier Ribeiro Antonino Carvalho: Contributed to study design, participated in data interpretation, and contributed to manuscript development. Fernando Guilherme Perazzo Costa: Conceived and supervised the study, contributed to study design, data analysis and interpretation, and critically revised the manuscript for important intellectual content.

## Disclosures

The authors declare that they have no known competing financial or personal interest that could have impact the outcome of the study reported in this paper.
